# RCS reduction using grounded multi-height multi-dielectrics metasurfaces

**DOI:** 10.1038/s41598-023-27853-4

**Published:** 2023-02-21

**Authors:** Maryam Heidari, Seyed Hassan Sedighy, Mohamad Khalaj Amirhosseini

**Affiliations:** 1 School of Electrical Engineering, Iran University of Science and Technology, Tehran, Iran; 2 School of Advanced Technologies, Iran University of Science and Technology, Tehran, Iran

**Keywords:** Aerospace engineering, Electrical and electronic engineering

## Abstract

In this paper, the PO method with the array theory are used to formulate the RCS of a grounded multi-height dielectric surface which can be employed in the design and optimization of a metasurface consisting of dielectric tiles with different heights and permittivities. The proposed closed form relations can be used instead of full wave simulation to design an optimized dielectric grounded metasurface, properly. Finally, three different RCS reducer metasurfaces are designed and optimized with three different dielectric tiles by using the proposed analytical relations. The results prove that the proposed ground dielectric metasurface achieve more than 10 dB RCS reduction at 4.4–16.3 GHz (114.9%) frequency range. This result proves the accuracy and effectiveness of the proposed analytical method which can be used in the RCS reducer metasurfaces design.

## Introduction

In the case of radar tracking, a target exposed to an incident wave acts as secondary antenna; where interaction of the transmitted wave with the target results in partial absorption of the received energy and conversion of the rest into scattered waves. The radar cross section (RCS) is a measure of target visibility. Therefore, the primary necessity of RCS calculation in concealment technology is target visibility reduction. In the other hand, accurate calculation of scattered fields requires a complete solution of the boundary value problem using Maxwell equations. But, accurate analytical solution exists for only a limited number of ideal bodies such as ones presented in^1,2^. To date, several numerical and asymptotic analytical techniques have been developed to predict a target RCS. Numerical methods such as moment method (MoM), finite difference time domain method (FDTD), fast multipolar method (FMM) and transmission line matrix (TLM) have been used to predict RCS of radar targets, also^3–11^. These methods are not dependent on geometry and can be used for any object with a desired shape. However, design and optimization of large electrical objects by using these numerical methods are time consuming and requires complex calculations, especially at high frequencies.

In addition to numerical methods, high frequency asymptotic methods, such as geometric optics (GO), physical optics (PO), uniform theory of diffraction (UTD) and physical theory of diffraction (PTD) have been used to formulate RCS^12^, also. These methods require less calculations compared with numerical methods and their simulation time is much shorter, consequently^13^. However, these methods have complex scattering field formulations for objects with complex shapes, which is more complicated in UTD rather than GO and PO methods^14^. The GO method is the fastest method among high frequency techniques, but has a relatively lower accuracy^14^. In other hands, the PO method gives much more accurate results compared to GO one and is an accepted method for formulating the scattered field of large electric objects^15,16^. Due to this relatively high accuracy, the PO method has been widely used for RCS formulations^17–19^. Heretofore, PO approximation is extensively used for PEC objects and structures covered with lossy dielectric materials^20–24^. Moreover, a rational design paradigm using quasibound states in the continuum and momentum analysis have been introduced in^25,26^ to analyze metasurfaces in the optical and near-infrared regime, also.

In this paper, the PO method along with the array theory are used to formulate the RCS of a grounded dielectric which can be employed in the design and optimization of a metasurface consisting of dielectric tiles with different heights and permittivities. These metasurfaces can be easily constructed and optimized to reduce the RCS of PEC targets. In more detail, analytical formulations are extracted to achieve RCS reduction of dielectric grounded tiles which can be used in the design and optimization of RCS reducer metasurfaces, effectively.

The rest of the paper is organized as follows: the scattering of an infinite grounded dielectric is formulated and validated in section “Infinite grounded dielectric slab Plane wave scattering”. Since the finite grounded plane is used in the proposed multi height dielectric metasurfaces, a finite plane approximation of the scattering formulation (discussed in section “Infinite grounded dielectric slab Plane wave scattering”) is required which is discussed in section “Plane wave scattering from a finite rectangular grounded dielectric surface”. The RCS of a finite dielectric metasurfaces is validated by simulation in section “Dielectric metasurface RCS simulation and verification”, also. Finally, three optimized metasurfaces composed of multi dielectric tiles are designed for RCS reduction by using the proposed analytical method.

## Infinite grounded dielectric slab Plane wave scattering

### Equivalation

**Figure 1:**
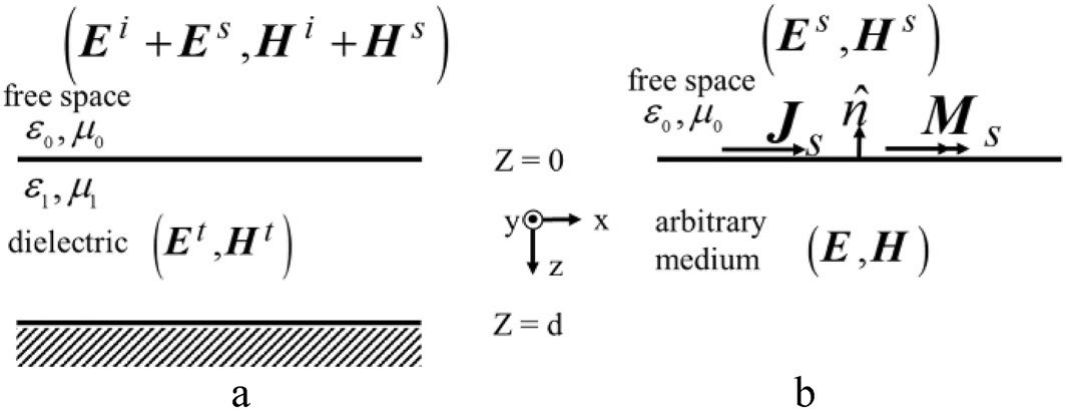
Employment of the surface equivalence theorem (**a**) main problem, (**b**) equivalent problem.

Using the surface equivalence theorem and assuming zero fields within the $z = 0$ closed surface (which is not the region of interest), the equivalent problem of Fig. 1a reduces to Fig. 1b with equivalent current densities equal to

1 $${\varvec{J}}_{s} = \left. {\hat{n} \times ({\varvec{H}}_{s} - {\varvec{H}})} \right|_{{{\varvec{H}} = 0}} = - \hat{z} \times {\varvec{H}}_{s}$$

2 $${\varvec{M}}_{s} = \left. { - \hat{n} \times ({\varvec{E}}_{s} - {\varvec{E}})} \right|_{{{\varvec{E}} = 0}} = \hat{z} \times {\varvec{E}}_{s}$$

**Figure 2:**
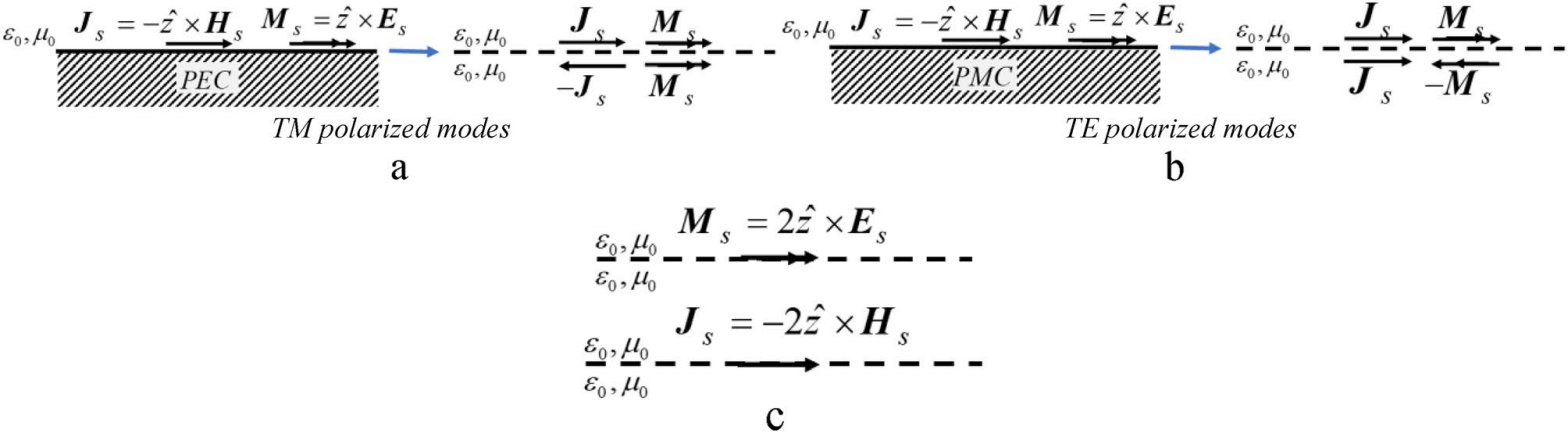
Equivalent models for scattering by infinite grounded dielectric slab (top for TE and bottom for TM). (**a**) Placement of a perfect conductor within the z = 0 closed surface. (**b**) Introduction of imaginary sources using of image theory. (**c**) Reduction after superposition of sources.

Now, placement of a PEC (or PMC) within the closed surface allows the use of image theory while the assumption of zero fields (which can be any value) is not violated. The radiation properties of the surface currents over the infinite flat PEC plane (Fig. 2a) can be obtained from the equivalent system depicted in Fig. 2b, that replaces actual sources by the imaginary ones on the side of the conductor while removing the conductor.

After superposition of sources (Fig. 2c) for TE and TM, equivalent currents become

3 $$TE:{\varvec{M}}_{s} = \left. {2(\hat{z} \times {\varvec{E}}_{s} } \right|_{z = 0} ) = - \hat{x}2R_{TE} E_{0} e^{{ - jk_{x} x}}$$

4 $$TM:{\varvec{J}}_{s} = \left. { - 2(\hat{z} \times {\varvec{H}}_{s} } \right|_{z = 0} ) = - \hat{x}2\frac{{R_{TM} E_{0} }}{{\eta_{0} }}e^{{ - jk_{x} x}}$$

### Validation

To validate the above results, it is appropriate to obtain the fields within $Z < 0$ region resulting from the surface currents relations expressed in (3) and (4) by using the radiation equations in an unbounded medium. For this purpose, the electric and magnetic potentials are desirable as

5 $$TE:{\varvec{A}} = 0,\,\,\,{\varvec{F}} = F_{x} \hat{x}$$

6 $$TM:{\varvec{A}} = A_{x} \hat{x},\,\,\,{\varvec{F}} = 0$$

By considering $\mathfrak{L} = \xi \sin \theta_{r} \cos \varphi_{r} + \eta \sin \theta_{r} \sin \varphi_{r}$, the electric and magnetic potential can be written as

7 $$\begin{aligned} F_{x} & = \frac{{e^{{ - jk_{0} r}} }}{{4\pi \varepsilon_{0}^{ - 1} r}}\int\limits_{S} {M_{x} } e^{{jk_{0} \mathfrak{L}}} dS \\ & = - R_{TE} E_{0} .\frac{{e^{{ - jk_{0} r}} }}{{2\pi \varepsilon_{0}^{ - 1} r}}\int\limits_{ - \infty }^{\infty } {\int\limits_{ - \infty }^{\infty } {e^{{ - j\xi (k_{x}^{i} - k_{0} \sin \theta_{r} \cos \varphi_{r} )}} e^{{j\eta k_{0} \sin \theta_{r} \sin \varphi_{r} }} d\xi d\eta } } \\ & = - R_{TE} E_{0} .\frac{{e^{{ - jk_{0} r}} }}{{\varepsilon_{0}^{ - 1} r}}.2\pi \delta (k_{x}^{i} - k_{x}^{r} )\delta (k_{y}^{r} ) \\ \end{aligned}$$

8 $$\begin{aligned} A_{x} & = \frac{{e^{{ - jk_{0} r}} }}{{4\pi \mu_{0}^{ - 1} r}}\int\limits_{S} {J_{x} } e^{{jk_{0} \mathfrak{L}}} dS \\ & = - \frac{{R_{TM} E_{0} }}{{\eta_{0} }}.\frac{{e^{{ - jk_{0} r}} }}{{2\pi \mu_{0}^{ - 1} r}}\int\limits_{ - \infty }^{\infty } {\int\limits_{ - \infty }^{\infty } {e^{{ - j\xi (k_{x}^{i} - k_{0} \sin \theta_{r} \cos \varphi_{r} )}} e^{{j\eta k_{0} \sin \theta_{r} \sin \varphi_{r} }} d\xi d\eta } } \\ & = - \frac{{R_{TM} E_{0} }}{{\eta_{0} }}.\frac{{e^{{ - jk_{0} r}} }}{{\mu_{0}^{ - 1} r}}.2\pi \delta (k_{x}^{i} - k_{x}^{r} )\delta (k_{y}^{r} ) \\ \end{aligned}$$

where $k_{x}^{r} = k_{0} \sin \theta \cos \varphi$ and $k_{y}^{r} = k_{0} \sin \theta \sin \varphi$. We have from identity (4-124) in^27^:

9 $$\frac{{e^{{ - jk_{0} r}} }}{r} = \frac{1}{2\pi j}\int\limits_{ - \infty }^{\infty } {\int\limits_{ - \infty }^{\infty } {\frac{{e^{{ - jz\sqrt {k_{0}^{2} - k_{x}^{r2} - k_{y}^{r2} } }} }}{{\sqrt {k_{0}^{2} - k_{x}^{r2} - k_{y}^{r2} } }}e^{{ - jk_{x}^{r} x}} e^{{ - jk_{y}^{r} y}} dk_{x}^{r} dk_{y}^{r} } }$$

By combining (7) and (8) with (9), one can find

10 $$F_{x} = - \frac{{R_{TE} E_{0} }}{{\varepsilon_{0}^{ - 1} }}.\frac{{e^{{jk_{z}^{r} z}} }}{{jk_{z}^{r} }}.e^{{ - jk_{x}^{i} x}}$$

11 $$A_{x} = - \frac{{R_{TM} E_{0} }}{{\mu_{0}^{ - 1} \eta_{0} }}.\frac{{e^{{jk_{z}^{r} z}} }}{{jk_{z}^{r} }}.e^{{ - jk_{x}^{i} x}}$$

which gives

12 $$\begin{aligned} k_{y}^{r} & = 0\,\, \Rightarrow \,\,\sin \varphi = 0\, \\ k_{x}^{r} & = k_{x}^{i} = k_{0} \sin \theta_{i} \\ k_{z}^{r} & = \sqrt {k_{0}^{2} - k_{x}^{r2} } = k_{0} \cos \theta_{i} = k_{z}^{i} \\ \end{aligned}$$

From the relations between fields and potentials, the following equations can be written as

13 $$H_{y} = \frac{1}{{\mu_{0} }}.\frac{{\partial A_{x} }}{\partial z} = - \frac{{R_{TM} E_{0} }}{{\eta_{0} }}.e^{{jk_{z}^{i} z}} .e^{{ - jk_{x}^{i} x}}$$

14 $$E_{y} = - \frac{1}{{\varepsilon_{0} }}.\frac{{\partial F_{x} }}{\partial z} = R_{TE} E_{0} .e^{{jk_{z}^{i} z}} .e^{{ - jk_{x}^{i} x}}$$

which are same as the scattered TE and TM fields obtained from the surface currents (3) and (4), respectively and prove the propped formulation, consequently.

## Plane wave scattering from a finite rectangular grounded dielectric surface

**Figure 3:**
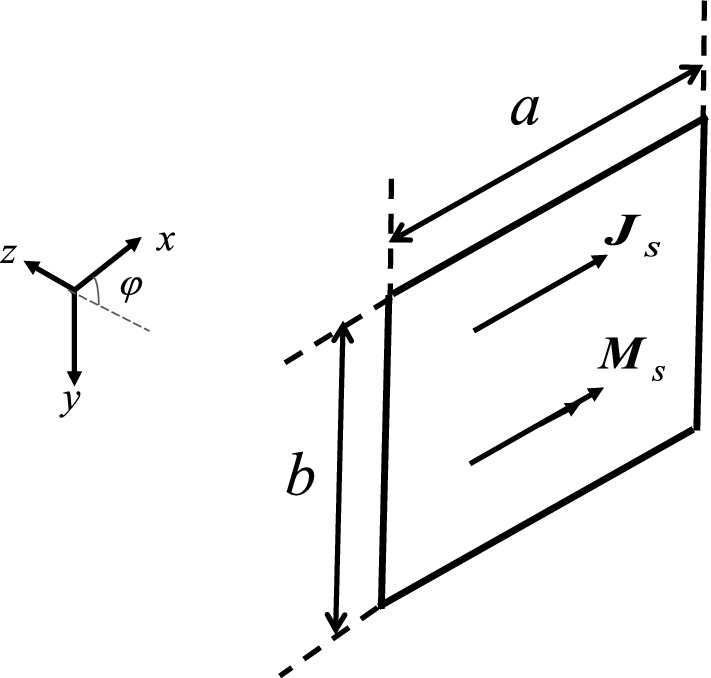
The equivalent problem of finite dielectric slab.

In order to obtain the scattering filed of the finite size grounded dielectric plate (shown in Fig. 3) based on the previously formulation (discussed in section “Infinite grounded dielectric slab Plane wave scattering”), it would be a good approximation if the radiation from the surface currents with the currents relations expressed in (3) and (4) can be obtained.

Therefore, the electric and magnetic vector potentials can be expressed as ($k_{x}^{i} = k_{x}$):

15 $$\begin{aligned} F_{x} & = - R_{TE} E_{0} .\frac{{e^{{ - jk_{0} r}} }}{{2\pi \varepsilon_{0}^{ - 1} r}}\int\limits_{{ - \frac{b}{2}}}^{\frac{b}{2}} {\int\limits_{{ - \frac{a}{2}}}^{\frac{a}{2}} {e^{{ - j\xi (k_{x} - k_{0} \sin \theta_{s} \cos \varphi_{s} )}} e^{{j\eta k_{0} \sin \theta_{s} \sin \varphi_{s} }} d\xi d\eta } } \\ & = - R_{TE} E_{0} .\frac{{e^{{ - jk_{0} r}} }}{{2\pi \varepsilon_{0}^{ - 1} r}}.\frac{a\sin (X)}{{^{X} }}.\frac{b\sin (Y)}{Y} \\ \end{aligned}$$

16 $$\begin{aligned} A_{x} & = - \frac{{R_{TM} E_{0} }}{{\eta_{0} }}.\frac{{e^{{ - jk_{0} r}} }}{{2\pi \mu_{0}^{ - 1} r}}\int\limits_{{ - \frac{b}{2}}}^{\frac{b}{2}} {\int\limits_{{ - \frac{a}{2}}}^{\frac{a}{2}} {e^{{ - j\xi (k_{x} - k_{0} \sin \theta_{s} \cos \varphi_{s} )}} e^{{j\eta k_{0} \sin \theta_{s} \sin \varphi_{s} }} d\xi d\eta } } \\ & = - \frac{{R_{TM} E_{0} }}{{\eta_{0} }}.\frac{{e^{{ - jk_{0} r}} }}{{2\pi \mu_{0}^{ - 1} r}}.\frac{a\sin (X)}{X}.\frac{b\sin (Y)}{Y} \\ \end{aligned}$$

where $$X = \frac{{k_{0} a}}{2}[\sin \theta_{i} - \sin \theta_{s} \cos \varphi_{s} ]$$ and $$Y = \frac{{k_{0} b}}{2}\sin \theta_{s} \sin \varphi_{s}$$.

The spherical components of potentials are also required for far-field relations as

17 $${\varvec{A}}_{T} = A_{\theta } \hat{1}_{\theta } + A_{\varphi } \hat{1}_{\varphi } ,\,\,A_{\theta } = \cos \theta_{s} \cos \varphi_{s} A_{x} \,\,,\,\,A_{\varphi } = - \sin \varphi_{s} A_{x}$$

18 $${\varvec{F}}_{T} = F_{\theta } \hat{1}_{\theta } + F_{\varphi } \hat{1}_{\varphi } ,\,\,F_{\theta } = \cos \theta_{s} \cos \varphi_{s} F_{x} \,\,\,,\,\,F_{\varphi } = - \sin \varphi_{s} F_{x}$$

As a result, scattered fields can be obtained as:

19 $${\varvec{H}}_{TE}^{s} = - j\omega {\varvec{F}}_{T} ,\,\,\,{\varvec{E}}_{TE}^{s} = j\omega \eta_{0} (\hat{1}_{r} \times {\varvec{F}}_{T} )$$

20 $$\begin{aligned} {\varvec{E}}_{TE}^{s} & = \, - j\omega \sqrt {\mu_{0} \varepsilon_{0} } . \\ & \;\;\;\left( {\cos \theta_{s} \cos \varphi_{s} \hat{1}_{\varphi } + \sin \varphi_{s} \hat{1}_{\theta } } \right)R_{TE} E_{0} .\frac{{e^{{ - jk_{0} r}} }}{2\pi r}.\frac{a\sin (X)}{{^{X} }}.\frac{b\sin (Y)}{Y} \\ \end{aligned}$$

21 $${\varvec{E}}_{TM}^{s} = - j\omega {\varvec{A}}_{T} ,\,\,\,{\varvec{H}}_{TM}^{s} = \frac{ - j\omega }{{\eta_{0} }}(\hat{1}_{r} \times {\varvec{A}}_{T} )$$

22 $$\begin{aligned} {\varvec{H}}_{TM}^{s} & = j\omega \varepsilon_{0} . \\ & \;\;\left( {\cos \theta_{s} \cos \varphi_{s} \hat{1}_{\varphi } + \sin \varphi_{s} \hat{1}_{\theta } } \right)R_{TM} E_{0} .\frac{{e^{{ - jk_{0} r}} }}{2\pi r}.\frac{a\sin (X)}{X}.\frac{b\sin (Y)}{Y} \\ \end{aligned}$$

Finally, the rectangular grounded dielectric surface RCS can be expressed as^26^:

23 $$\begin{aligned} TE:\,\,\,\sigma_{3 - D} & = \mathop {\lim }\limits_{r \to \infty } \left[ {4\pi r^{2} \frac{{\left| {{\varvec{E}}^{s} } \right|^{2} }}{{\left| {{\varvec{E}}^{i} } \right|^{2} }}} \right] \\ & \;\; = 4\pi \left( {\cos^{2} \theta_{s} \cos^{2} \varphi_{s} + \sin^{2} \varphi_{s} } \right)\left( {R_{TE} .\frac{ab}{\lambda }} \right)^{2} .\left( {\frac{\sin (X)}{{^{X} }}.\frac{\sin (Y)}{Y}} \right)^{2} \\ \end{aligned}$$

24 $$\begin{aligned} TM:\,\,\,\sigma_{3 - D} & = \mathop {\lim }\limits_{r \to \infty } \left[ {4\pi r^{2} \frac{{\left| {{\varvec{H}}^{s} } \right|^{2} }}{{\left| {{\varvec{H}}^{i} } \right|^{2} }}} \right] \\ & = 4\pi \left( {\cos^{2} \theta_{s} \cos^{2} \varphi_{s} + \sin^{2} \varphi_{s} } \right)\left( {R_{TM} .\frac{ab}{\lambda }} \right)^{2} .\left( {\frac{\sin (X)}{X}.\frac{\sin (Y)}{Y}} \right)^{2} \\ \end{aligned}$$

## Dielectric metasurface RCS simulation and verification

Here, a finite grounded dielectric metasurface is considered to validate the proposed analytical method. This surface is considered as a grounded FR4 dielectric with 3 mm thickness and dimensions of 144 mm × 114 mm.

**Figure 4:**
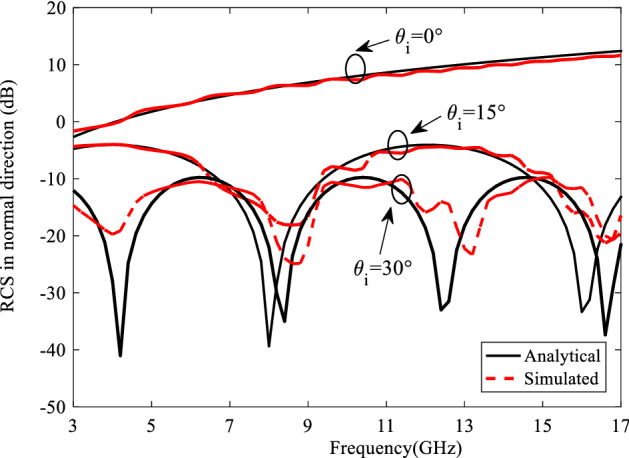
The comparison of analytical and simulated RCS of finite grounded dielectric in normal incidence.

The comparison of analytical RCS using obtained from (23) and (24) with the full wave simulation results is given in Fig. 4. Notice that all of the full wave simulations are done with CST Microwave Studio. Also, the surface is illuminated by the incident wave in normal and oblique directions, where transmitter is located in a normal direction respect to the surface.

**Figure 5:**
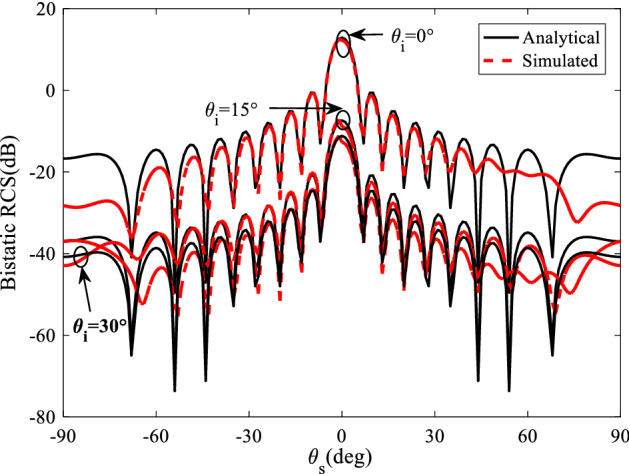
The comparison of analytical and simulated bistatic results at 18 GHz in the plane that contains the maximum scattered field ($\varphi_{s} = {\pi \mathord{\left/ {\vphantom {\pi 2}} \right. \kern-0pt} 2}$) for $\theta_{i} = 0^{ \circ } ,15^{ \circ } ,30^{ \circ }$.

A comparison of analytical and simulated bistatic results is depicted in Fig. 5 at 18 GHz in the plane that contains the maximum scattered field ($\varphi_{s} = {\pi \mathord{\left/ {\vphantom {\pi 2}} \right. \kern-0pt} 2}$) for $\theta_{i} = 0^{ \circ } ,15^{ \circ } ,30^{ \circ }$. These results confirm the agreement of the analysis with the simulation which prove the proposed analytical relations.

If we consider zero thickness for the dielectric layer, we will have the scattering from the rectangular PEC plate without dielectric, in which the reflection and RCS are as follows:

25 $$R_{TE/TM} = \frac{{ - p_{TE/TM} + j\tan 0}}{{p_{TE/TM} + j\tan 0}} = - 1\,\,\,$$

26 $$\begin{aligned} TE\& TM:\,\,\,\sigma_{3 - D} & = \mathop {\lim }\limits_{r \to \infty } \left[ {4\pi r^{2} \frac{{\left| {{\varvec{E}}^{s} } \right|^{2} }}{{\left| {{\varvec{E}}^{i} } \right|^{2} }}} \right] \\ & = 4\pi \left( {\cos^{2} \theta_{s} \cos^{2} \varphi_{s} + \sin^{2} \varphi_{s} } \right)\left( {\frac{ab}{\lambda }} \right)^{2} .\left( {\frac{\sin (X)}{{^{X} }}.\frac{\sin (Y)}{Y}} \right)^{2} \\ \end{aligned}$$

which is same as the one obtained in^28^.

From the Eqs. (23), (24) and (26), one can obtain the RCS reduction in the case where the PEC plate is coated with dielectric compared to the PEC plate without dielectric coating as

27 $$RCS{\text{Re}} duction_{TE/TM} = 20\log R_{TE/TM}$$

## Grounded multi-height multi-dielectric metasurface design

**Figure 6:**
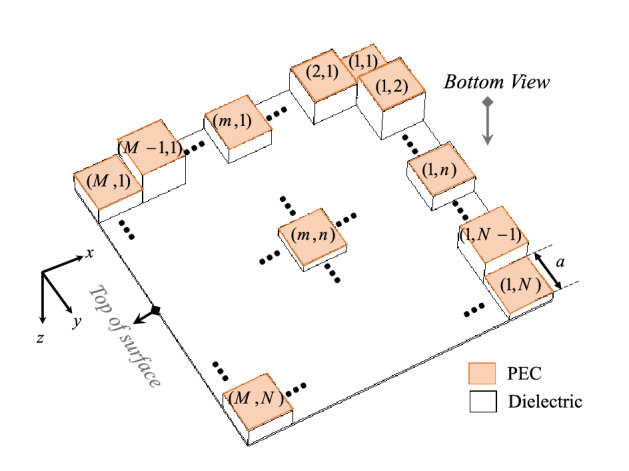
Bottom view of 2D metasurface composed of different grounded dielectrics tiles (M × N tiles) with different $\varepsilon_{r}$ and heights.

The proposed RCS method for analytical calculation of RCS reduction in the previous section can be used for analytical low scattering metasurface design, properly. For a 2D metasurface composed of different grounded dielectrics tiles with different relative permittivity ($\varepsilon_{r}$) and heights (shown in Fig. 6 from bottom view), the scattered fields can be expressed by using the theory of arrays as^29^

28 $$\begin{aligned} {\varvec{E}}_{TE}^{s} & = \, - j\omega \sqrt {\mu_{0} \varepsilon_{0} } a^{2} \left( {\cos \theta_{s} \cos \varphi_{s} \hat{1}_{\varphi } + \sin \varphi_{s} \hat{1}_{\theta } } \right)E_{0} . \\ & \;\;\;\frac{{e^{{ - jk_{0} r}} }}{2\pi r}.\frac{\sin (X)}{{^{X} }}.\frac{\sin (Y)}{Y}\mathop \sum \limits_{m,n} R_{TE(m,n)} e^{{j\psi_{mn} }} \\ \end{aligned}$$

29 $$\begin{aligned} {\varvec{H}}_{TM}^{s} & = j\omega \varepsilon_{0} a^{2} \left( {\cos \theta_{s} \cos \varphi_{s} \hat{1}_{\varphi } + \sin \varphi_{s} \hat{1}_{\theta } } \right)E_{0} . \\ & \;\;\frac{{e^{{ - jk_{0} r}} }}{2\pi r}.\frac{\sin (X)}{X}.\frac{\sin (Y)}{Y}\mathop \sum \limits_{m,n} R_{TM(m,n)} e^{{j\psi_{mn} }} \\ \end{aligned}$$

30 $$\psi_{mn} = ka\left. {\left\{ {m\left( {cos\theta_{i} sin\phi_{i} - cos\theta_{sc} sin\phi_{sc} } \right) + n\left( {sin\theta_{i} - sin\theta_{sc} } \right)} \right.} \right\}$$

where *a* is the width of square tiles.

Therefore, RCS of this metasurface which is composed of different tiles can be written as

31 $$\begin{aligned} \sigma_{3 - D(TE/TM)} & = 4\pi \left( {\cos^{2} \theta_{s} \cos^{2} \varphi_{s} + \sin^{2} \varphi_{s} } \right) \\ & \;\;\; \cdot \left( {\left| {\mathop \sum \limits_{m,n} R_{TE/TM(m,n)} e^{{j\psi_{mn} }} } \right|\frac{{a^{2} }}{\lambda }} \right)^{2} .\left( {\frac{\sin (X)}{{^{X} }}.\frac{\sin (Y)}{Y}} \right)^{2} \\ \end{aligned}$$

The reduction value of RCS as for the aforementioned array compared to the same size uncoated PEC can be obtained in dB as

32 $$RCS{\text{Re}} duction_{TE/TM} = 20\log \left| {\mathop \sum \limits_{m,n} \frac{{R_{TE/TM(m,n)} }}{MN}e^{{j\psi_{mn} }} } \right|$$

where *M* and *N* are the number of tiles in *x* and *y* directions, respectively. Notice that, this relation achieve RCS reduction of a grounded dielectric, analytically with good enough accuracy rather than the full wave ones. In fact, one can uses this relation in RCS reducer metasruface design and optimization process instead of using full wave simulation, effectively.

As an example, a three tile metasurface is considered by using three different dielectric tiles with periodic arrangement (*M* = *N* = 3) where (1,1)-*th*, (2,3)*-th*, (3,2)-*th* tiles are same called tile I, (1,2)-*th*, (2,1)*-th*, (3,3)-*th* tiles are same called tile II, and (1,3)-*th*, (2,2)*-th*, (3,1)-*th* tiles are same called tile III as shown in Figs. 8, 9 and 10.

By considering the conventional specular direction ($\phi_{sc} = \phi_{i}$ and $\theta_{sc} = \theta_{i}$), (30) becomes $\psi_{mn} = 0$, and $R_{TE/TM(m,n)} = e^{{j\phi (\varepsilon_{r(m,n)} ,d_{(m,n)} )}}$ for (m,n)-*th* tile by using (A12) (Supplementary file). According to (32), the related specular RCS reduction of the three tile metasurface compared with a PEC surface with same size, can be expressed as follows

33 $$RCS\,Reduction_{3} { } = { }20{ } \times \log_{10} \left| {\frac{1}{3} \times \left( {e^{{j\phi (\varepsilon_{rI} ,d_{I} )}} + e^{{j\phi (\varepsilon_{rII} ,d_{II} )}} + e^{{j\phi (\varepsilon_{rIII} ,d_{III} )}} } \right)} \right|$$

And after some simplifications, this relation can be expressed as

34 $$RCS\,Reduction_{3} { } = { }20{ } \times \log_{10} \left| {\frac{1}{3} \times \left( {1 + e^{{j\phi_{1} }} + e^{{j\phi_{2} }} } \right)} \right|$$

**Figure 7:**
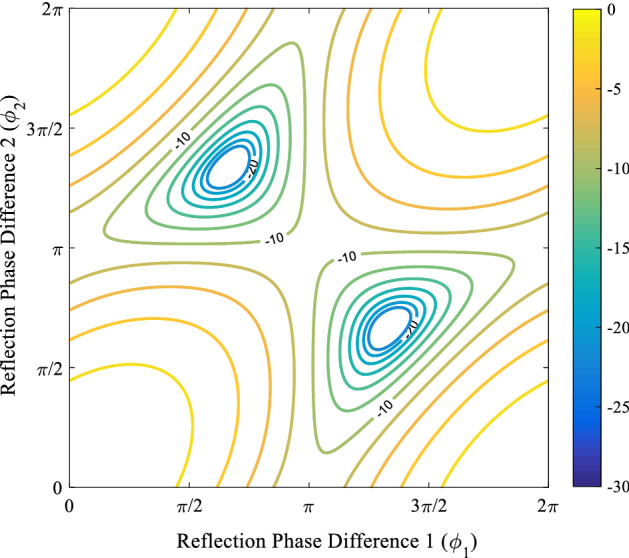
Depiction of the intersection of the 3D surfaces of (33) and $RCS\,Reduction_{3} { } = cte$ in the $\phi_{1} \phi_{2}$ plane.

One of the tiles here is chosen to have the reference phase ($\phi (\varepsilon_{rI} ,d_{I} )$) and the two remaining tiles take relative phase with respect to that of the reference ($\phi_{1} = \phi (\varepsilon_{rII} ,d_{II} ) - \phi (\varepsilon_{rI} ,d_{I} )\& \phi_{2} = \phi (\varepsilon_{rIII} ,d_{III} ) - \phi (\varepsilon_{rI} ,d_{I} )$). The RCS reduction contour of this surface versus $\Phi_{1}$ and $\Phi_{2}$ is shown in Fig. 7. In more details, the phase difference between the building tiles should be inside the corresponding closed curve in this figure to achieve values (or better) of determined RCS reduction.

**Figure 8:**
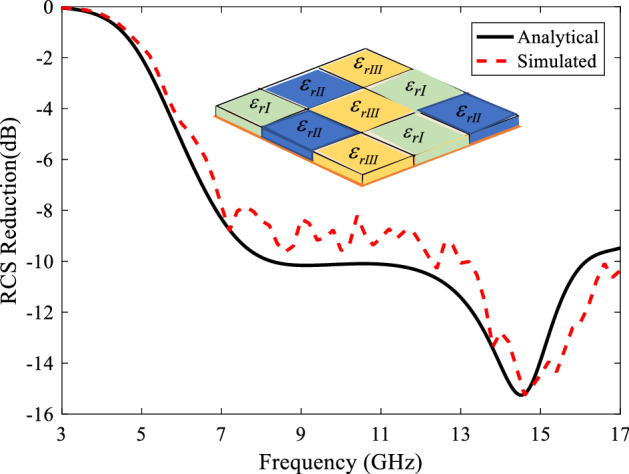
Analytical RCS reduction of Case I metasurface compared with the full-wave simulation one.

***Optimization***. The Surrogate optimization algorithm^30^ is used to optimize the height and relative permittivity of three tiles in order to achieve a wide 10-dB RCS reduction bandwidth ($C = 10$). The Surrogate searches space of dielectric parameters ($1 \le \varepsilon_{rn} \le 4.3$ and $0.2\;{\text{mm}} \le h_{n} \le 10\;{\text{mm}}$ where $n \in \left\{ {tileI,tileII,tileIII} \right\}$) to find the best values of these three tiles which $\phi_{1}^{i}$ and $\phi_{2}^{i}$ of them falls inside the corresponding desired contour in Fig. 8 for all bandwidth frequencies, where $i \in [f_{\min } ,f_{\max } ]$.

In fact, the below cost function should be minimized by this optimization method.

35 $${\text{Cos}} t = \min \sum\limits_{{i \in [f_{\min } ,f_{\max } ]}} {\left[ {\sqrt {\frac{{(\phi_{1}^{i} - \phi_{c1}^{i} )^{2} + (\phi_{2}^{i} - \phi_{c2}^{i} )^{2} }}{{\phi_{c1}^{i2} + \phi_{c2}^{i2} }}} } \right]}$$

where $(\phi_{c1}^{i} ,\phi_{c2}^{i} )$ indicates the closest point on the desired curve to the phase difference point $(\phi_{1}^{i} ,\phi_{2}^{i} )$ of tiles at the i-th frequency point.

Here, we consider three different RCS reducer metasurfaces to evaluate the proposed design method, properly. Notice that the tiles are considered as 100 mm × 100 mm to achieve periodicity at each tile.

***Case I***. The first case is dielectric tiles with same heights and different relative permittivities. To find the best three tiles, the optimizer should find the four variables $(\varepsilon_{rI} ,\varepsilon_{rII} ,\varepsilon_{rIII} ,h)$ to achieve the most 10-dB RCS reduction frequency bandwidth. The arrangement of these tiles in the metasurface structure can be seen in Fig. 8, where different colors are used for different permittivities. The optimized dielectric constant and thickness values are obtained as $\varepsilon_{rI} = 2.9$, $\varepsilon_{rII} = 4.3$, $\varepsilon_{rIII} = 1$, $h = 7.21\;{\text{mm}}$. In this figure, the analytical RCS reduction of the metasurface is compared with the full-wave one. Such a dielectric array configuration reduces RCS of the PEC plate about 10 dB at 8.2–16.2 GHz (B.W. = 65.6%).

**Figure 9:**
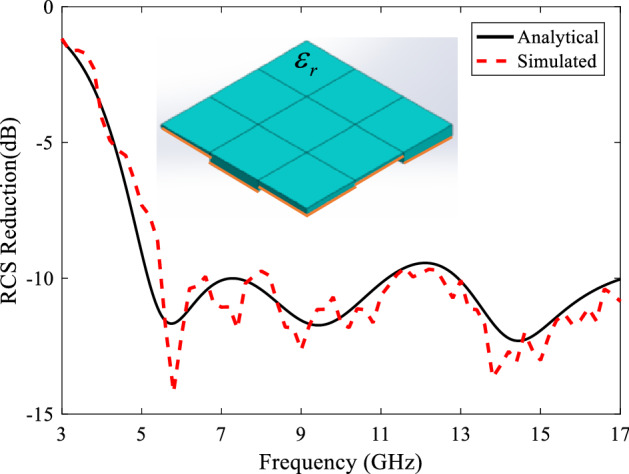
Analytical RCS reduction of Case II metasurface compared with the full-wave simulation one.

***Case II***. In this case, dielectrics with same relative permittivity and different heights are considered. To find the best three tiles, the optimizer should find the four variables $(h_{I} ,h_{II} ,h_{III} ,\varepsilon_{r} )$ to achieve the most 10-dB RCS reduction frequency bandwidth. The arrangement of these tiles can be seen in Fig. 9. The optimized dielectric constant and thickness values are obtained as $\varepsilon_{r} = 2.69$, $h_{I} = 2.55\;{\text{mm}}$, $h_{II} = 10\;{\text{mm}}$, $h_{III} = 5.27\;{\text{mm}}$. In this figure, the analytical RCS reduction of the metasurface is compared with the full-wave one. Such a dielectric array configuration reduces RCS of the PEC plate by about 10 dB at 5.1–17 GHz (B.W. = 107%).

**Figure 10:**
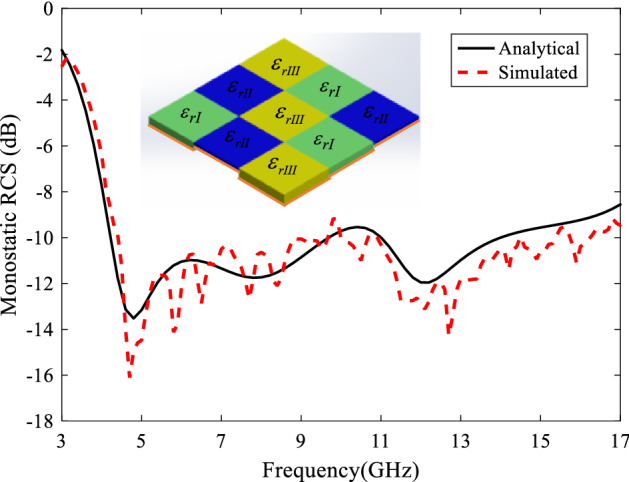
Analytical RCS reduction of Case III metasurface compared with the full-wave simulation one.

***Case III***. The third case is dielectrics tiles with different heights and relative permittivities. To find the best three tiles, the optimizer must find the six variables $(h_{I} ,h_{II} ,h_{III} ,\varepsilon_{rI} ,\varepsilon_{rII} ,\varepsilon_{rIII} )$ to achieve the most 10-dB RCS reduction frequency bandwidth. The arrangement of these tiles can be seen in Fig. 10 where different colors are used for different permittivities. The optimized dielectric constant and thickness values are obtained as $\varepsilon_{rI} = 1.62$, $h_{I} = 8.26$, $\varepsilon_{rII} = 2.6$, $h_{II} = 3.02$,$\varepsilon_{rIII} = 3.83$,$h_{III} = 10$. In this figure, the analytical RCS reduction of the metasurface is compared with the full-wave one. The good agreement between these two results proves the proposed method, also. Such a dielectric array configuration reduces the RCS of PEC plate by about 10 dB at 4.4–16.3 GHz (B.W. = 114.9%). Notice that negligible difference between the simulation and analysis results is due to the approximation used for the surface currents at the dielectric boundary.

## Conclusion

In this paper, PO approximation was used to solve the scattering problem of grounded dielectric, and its results were proved to be consistent with the results of full wave simulation CST software. Therefore, the validity of this approximation for dielectric metasurface design has been confirmed. Finally, it was shown that a combination of several different dielectrics tiles with different specifications (height and relative permitivity) can be used to minimize RCS in a broadband bandwidth.

## Supplementary Information

Supplementary Information.

## Data Availability

The datasets used and/or analysed during the current study available from the corresponding author on reasonable request.
