# Supplementary material for: RCS reduction using grounded multi-height multi-dielectrics metasurfaces
Source: Sci Rep. 2023 Feb 21;13:3069. doi: 10.1038/s41598-023-27853-4 (PMC9944268; doi:10.1038/s41598-023-27853-4)
Supplement: Supplementary file 1 — Supplementary Information. [file 41598_2023_27853_MOESM1_ESM.docx]

Appendix

## Scattering formulation

By assuming $e^{j\omega t}$ time dependence, and noting that the problem is symmetric in y (no y variations); $k_{y}=0$ electromagnetic fields can be obtained as follows.

Fig. A1. Plane wave scattering by infinite grounded dielectric slab

The fields components in each region of $n=0, 1$ (0 indicates free space and 1 indicates dielectric) are the sum of the outgoing (+) and incoming (-) plane waves, which in the $TE^{z}$case are displayed as follows:

$E_{yn}=(E_{n}^{+}e^{-jk_{zn}z}+E_{n}^{-}e^{jk_{zn}z})e^{-jk_{xn}x}$ (A1)

$H_{xn}=-\frac{jk_{zn}}{\hat{Z}_{n}}(E_{n}^{+}e^{-jk_{zn}z}-E_{n}^{-}e^{jk_{zn}z})e^{-jk_{xn}x}$ (A2)

$H_{zn}=\frac{jk_{xn}}{\hat{Z}_{n}}(E_{n}^{+}e^{-jk_{zn}z}+E_{n}^{-}e^{jk_{zn}z})e^{-jk_{xn}x}$ (A3)

In which $\hat{Z}_{n}=j\omega\mu_{n}$. Also, in the $TM^{z}$case:

$H_{yn}=(H_{n}^{+}e^{-jk_{zn}z}-H_{n}^{-}e^{jk_{zn}z})e^{-jk_{xn}x}$ (A4)

$E_{xn}=\frac{jk_{zn}}{\hat{Y}_{n}}(H_{n}^{+}e^{-jk_{zn}z}+H_{n}^{-}e^{jk_{zn}z})e^{-jk_{xn}x}$ (A5)

$E_{zn}=-\frac{jk_{xn}}{\hat{Y}_{n}}(H_{n}^{+}e^{-jk_{zn}z}-H_{n}^{-}e^{jk_{zn}z})e^{-jk_{xn}x}$ (A6)

where $\hat{Y}_{n}=j\omega\varepsilon_{n}$ ,and $k_{zn}^{2}+k_{xn}^{2}=k_{n}^{2}=\omega^{2}\mu_{n}\varepsilon_{n}$.

The tangential components of the fields must be continuous across the $z=0$ boundary and the tangential component of electric field must be zero across the $z=d$ boundary. By putting$E_{0}^{+}=E_{0}$,$E_{0}^{-}=R_{TE/TM}E_{0}$ , $H_{0}^{+}=E_{0}/\eta_{0}$ , $H_{0}^{-}=R_{TE/TM}E_{0}/\eta_{0}$, the boundary condition is written as

$k_{x0}=k_{x1}=k_{x}$ (A7)

$TE^{z}\left\{\begin{matrix} E_{0}(1+R_{TE})=E_{1}^{+}+E_{1}^{-} \\ -\frac{jk_{z0}}{\hat{Z}_{0}}E_{0}(1-R_{TE})=-\frac{jk_{z1}}{\hat{Z}_{1}}(E_{1}^{+}-E_{1}^{-}) \\ E_{1}^{+}e^{-jk_{z1}d}+E_{1}^{-}e^{jk_{z1}d}=0 \end{matrix}\right.$ (A8)

$TM^{z}\left\{\begin{matrix} \frac{E_{0}}{\eta_{0}}(1-R_{TM})=H_{1}^{+}-H_{1}^{-} \\ \frac{jk_{z0}}{\hat{Y}_{0}}\frac{E_{0}}{\eta_{0}}(1+R_{TM})=\frac{jk_{z1}}{\hat{Y}_{1}}(H_{1}^{+}+H_{1}^{-}) \\ H_{1}^{+}e^{-jk_{z1}d}+H_{1}^{-}e^{jk_{z1}d}=0 \end{matrix}\right.$ (A9)

By solving above system of equations we find:

(A10)

where

(A11)

The phasor representation of (A10) will be as follows:

(A12)
